# ACR70-disease activity score remission achievement from switches between all the available biological agents in rheumatoid arthritis: a systematic review of the literature

**DOI:** 10.1186/ar2848

**Published:** 2009-11-03

**Authors:** Stefano Alivernini, Antonella Laria, Elisa Gremese, Angelo Zoli, Gianfranco Ferraccioli

**Affiliations:** 1 Division of Rheumatology, School of Medicine, Catholic University of the Sacred Heart, Largo F Vito 1, 00168 Rome, Italy

## Abstract

**Introduction:**

The aim of our analysis was to compare the gaining of a major response (disease activity score [DAS] remission or American College of Rheumatology 70% improvement criteria [ACR70]) by switching between all the available biological therapies in rheumatoid arthritis.

**Methods:**

A systematic review was performed including studies, published before December 2008, in which a second biological agent was used and clinical outcomes were evaluated after a first biological failure.

**Results:**

Nine articles were included. Switching from etanercept and/or infliximab to adalimumab is effective with an ACR70 response ranging from 5% to 33%. Rituximab may be slightly more effective than switching to a second anti-tumor necrosis factor-alpha (anti-TNFα), reaching an ACR70 or DAS remission response in 12% and 9%, respectively. Clinical trials confirmed the efficacy in switching to abatacept (gain of effect 10.2%). Tocilizumab allows DAS28 (DAS using 28 joint counts) remission in 30.1% but ACR70 only in 12.4% of patients refractory to anti-TNFα.

**Conclusions:**

The efficacy of a second biological agent, irrespective of the mode of action, in reaching an ACR70 or DAS remission after a first biologic is observed from 5% to 15% and from 9% to 15.4%, respectively (except in two studies).

## Introduction

Three anti-tumor necrosis factor-alpha (anti-TNFα) therapies are approved for rheumatoid arthritis (RA) by the US Food and Drug Administration: infliximab (Remicade^®^), adalimumab (Humira^®^), and etanercept (Enbrel^®^). Two more will come soon (certolizumab pegol and golimumab). Although similarities clearly predominate when comparing the three available anti-TNFα agents, a number of clinical differences in efficacy or safety have been noted [1,2]. First, the half-lives - 3 days for etanercept, 10 days for infliximab, and 13 days for adalimumab - may translate into differences in the duration of TNFα neutralization [2]. Also, the two monoclonal antibodies, infliximab and adalimumab, have very strong affinity for TNFα, increasing the percentage of neutralized TNFα molecules. In addition, the complexes formed when monomeric and trimeric soluble and membrane-associated TNFα molecules bind to the anti-TNFα agent are far more stable with infliximab and adalimumab than with etanercept. Finally, the monoclonal antibodies are highly specific for TNFα, whether soluble or at the membrane level, whereas etanercept binds to lymphotoxin-α in addition to soluble TNFα, leading to the control of another possible pathogenetic pathway. Soluble TNFα binds to the fusion protein, becoming unable to act on its cellular receptor. Thus, etanercept has a buffering effect on TNFα, and this effect is probably reversible and does not result in permanent elimination of TNFα molecules. Furthermore, binding of etanercept to membrane-associated TNFα does not cause cell lysis. Infliximab and adalimumab can bind two soluble or membrane-associated TNFα molecules, forming a stable and long-lasting complex and causing cell lysis (for example, macrophages and some T-cell subsets) or cell function impairments [2]. These differences may influence the risk of immune response impairment and the ability to ward off infections, explaining the greater risk of tuberculosis with infliximab and adalimumab than with etanercept. Immunogenicity seems extremely weak for etanercept and adalimumab but higher for infliximab, inducing antibodies to its murine component (human anti-murine antibodies, or HAMA) and leading to allergic reactions and the often-seen escape phenomenon [2]. All of these data have led physicians to treat RA patients who experience treatment failure with one anti-TNFα agent (due to either inefficacy or toxicity) by switching to a second anti-TNFα agent, although the clear-cut benefits of switching are unknown because no controlled trial has ever been conducted.

Rituximab, or anti-CD20, is an antibody used in RA, whereas abatacept is a dimeric fusion soluble protein made of the extracellular part of CTLA-4 present on T cells and Fc of IgG1. It links CD80/86 on antigen-presenting cells with a higher affinity than CD28, thus preventing the costimulation. Tocilizumab is a humanized antibody that links both soluble and membranous interleukin-6 receptor. The differences in the mechanism of action should allow clinicians to rescue patients not fully responding to a TNFα blocker since a different pathway is targeted; however, a definite analysis of the gain of effect in terms of disease activity score (DAS) remission or of an American College of Rheumatology 70% improvement criteria (ACR70) response - that clearly allows clinicians to identify the crucial pathway alternative to TNFα - has not been provided. The aim of this study was to investigate the evidence in the literature about the efficacy of switching between different biologics in RA patients.

## Materials and methods

We performed a search on MEDLINE, EMBASE, and the Cochrane Library from inception to December 2008 to identify all of the available articles. The terms we used were 'arthritis', 'rheumatoid', 'biological agents (infliximab, etanercept, adalimumab, rituximab, anakinra, abatacept, tocilizumab)', 'switch or switching', 'randomized controlled trials', 'multicenter studies', 'clinical trials phase II', 'clinical trials phase III', and 'clinical trials phase IV'. We searched even in the abstract databases of both the European League Against Rheumatism (EULAR) and the ACR from 1996 to the present in order to identify unpublished studies. Articles were selected by applying predefined inclusion and exclusion criteria, and their methodological quality was graded according to the levels of evidence of the Centre for Evidence-Based Medicine (Oxford, UK) [3].

The language of the paper was not restricted. References of the studies were analyzed to find any study that was not included in the electronic databases. A study was included in the systematic extraction of the data if (a) it was published before December 2008, (b) it was about patients with RA, (c) a biological agent was used and failed according to the clinical response (DAS using 28 joint counts [DAS28] of greater than 2.6 or EULAR criteria for poor responders), and (d) a second biological agent was used and clinical outcomes were evaluated. The ACR responses (ACR20, ACR50, and ACR70) were used as efficacy parameters. During the writing process, only articles concerning major response, as ACR70 or the DAS showing disease remission, were included. The extraction of the data was conducted independently by two investigators.

## Results

**Figure 1:**
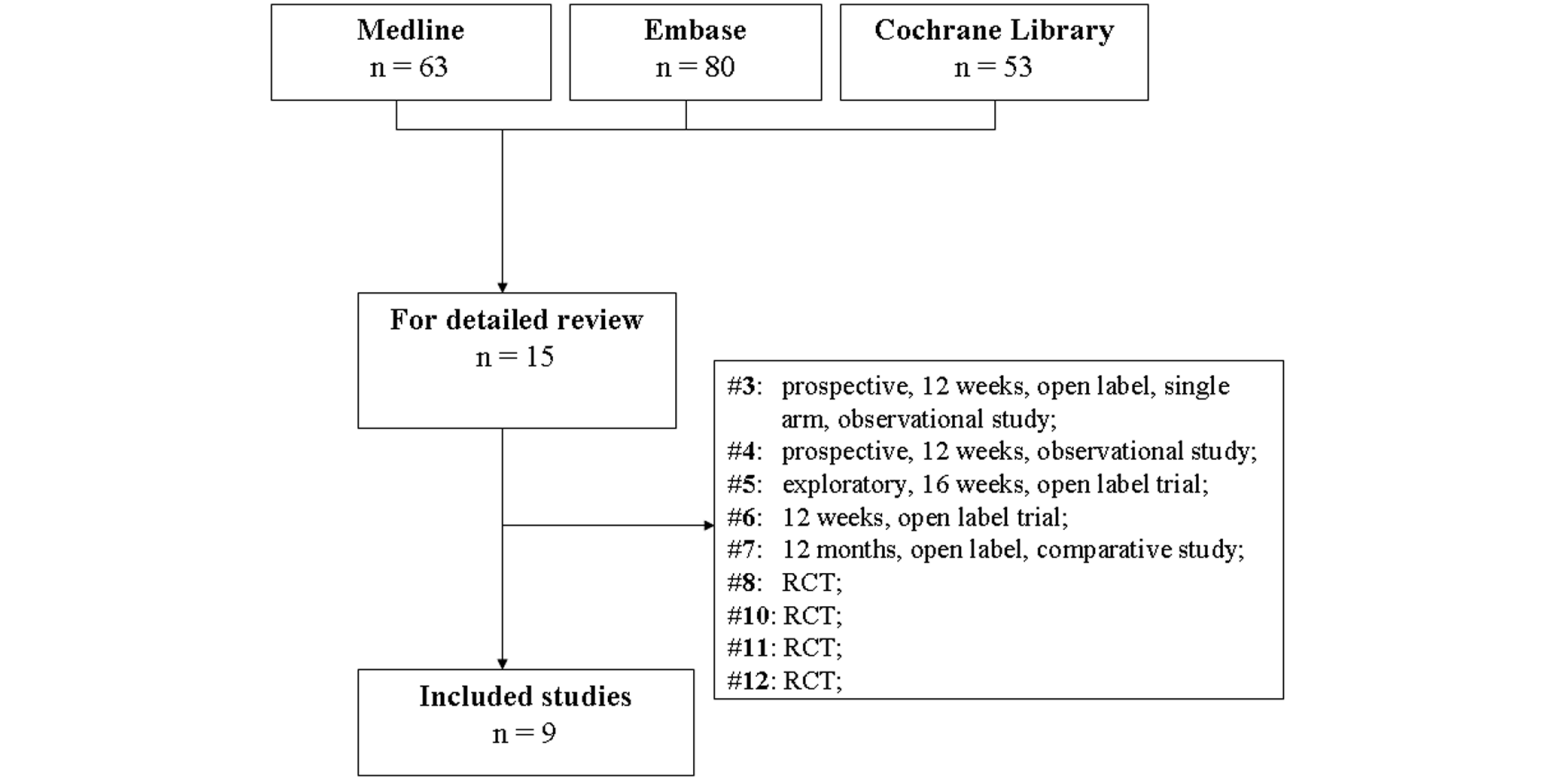
Search strategy tree. RCT, randomized controlled trial.

After the systematic literature research, nine articles fulfilled the inclusion criteria and were included in the review. All of the articles were divided according to the biological agent failed and switched (Figure 1 and Table 1).

**Table 1:** Gain of a major response by switching between different available biological agents in rheumatoid arthritis

	Results								
	
Study	Number of patients	Switch type	ACR20	ACR50	ACR70	DAS44 <1.6 or DAS28 <2.6	Δ^DAS ^*P *< 0.05	Evidence level^a^	Strength^a^
Anti-TNFα blockers									
[3]	25	IFX → ETA	64%	23%	5%	-	-	3b	B
[4]	95	IFX → ETA	38%	24%	15%	-	6.46 → 4.97	3b	B
[5]	28	ETA → IFX	62%	30.7%	-	15.4%	5.2 → 4	3b	B
[6]	6,610	ETA/IFX → ADA	60%	33%	13%	12%	31% (-1.9 ± 1.4)	2b	B
[7]	25	IFX → ADA	75%	50%	33%	-	5.6 → 3.2	3b	B

Anti-CD20									
[8]	311	Anti-TNFα → RTX	51%	27%	12%	9%	15% (Δ^DAS ^> 1.2)	1b	A

SR CTLA-4									
[10]	391	Anti-TNFα → ABA	50.4%	20.3%	10.2%	10.0%	-	1b	A
[11]	1,046	Anti-TNFα → ABA	-	-	-	13.0%	56.1% (-2.0)	1b	A

Interleukin-6R inhibitor									
[12]	499	Anti-TNFα → TOC	50.0%	28.8%	12.4%	30.1%	-	1b	A

^a^According to the levels of evidence of the Centre for Evidence-Based Medicine (Oxford, UK). Δ^DAS ^*P *<0.05, significant difference in disease activity score value before and after switching; ABA, abatacept; ACR20, American College of Rheumatology 20% improvement criteria; ACR50, American College of Rheumatology 50% improvement criteria; ACR70, American College of Rheumatology 70% improvement criteria; ADA, adalimumab; anti-TNFα, anti-tumor necrosis factor-alpha; DAS28, disease activity score using 28 joint counts; DAS44, disease activity score using 44 joint counts; ETA, etanercept; IFX, infliximab; RTX, rituximab; TOC, tocilizumab (8 mg/kg group).

### Rate of efficacy (gaining of major responses) in etanercept-treated patients who failed treatment with infliximab

In a prospective, 12-week, open-label, single-arm, observational study, Haraoui and colleagues [4] showed the efficacy of etanercept when infliximab had been ineffective. Twenty-five RA patients were enrolled; 18 of them discontinued infliximab because of lack of efficacy, and 22 completed 12 weeks of etanercept treatment. After 12 weeks of follow-up, 64% patients achieved at least an ACR20 response and 5% achieved an ACR70 response. Fifty-nine percent of patients experienced improvement in Health Assessment Questionnaire (HAQ) score (with a decrease of at least 0.22 in HAQ score) [4] (3b level of Oxford evidence).

Buch and colleagues [5] analyzed 95 RA patients who failed infliximab and methotrexate treatment and switched to etanercept. Thirty-four patients never achieved a response to infliximab (primary non-responders), 38 had an initial response to infliximab but relapsed (secondary non-responders), and 23 had side effects. After 12 weeks, 38% of patients achieved an ACR20 response whereas 24% and 15% achieved ACR50 and ACR70 responses, respectively. In the primary non-responder group, 42%, 30%, and 15% of patients achieved ACR20, ACR50, and ACR70 responses, respectively; the rates for the secondary non-responder group were 34%, 21%, and 14%, respectively. Similar results were obtained even comparing a moderate or good EULAR score (67% of primary and 56% of secondary infliximab failures) and DAS28 reduction [5] (3b level of Oxford evidence).

### Rate of efficacy (gaining major responses) in infliximab-treated patients who failed treatment with etanercept

In a randomized, open-label, clinical trial conducted by Furst and colleagues [6], 28 RA patients with an inadequate response to etanercept were randomly assigned to discontinue etanercept and receive infliximab (group 1) or to continue etanercept (group 2). At week 16, 62% of infliximab-treated patients achieved an ACR20 response compared with 29% of etanercept-treated patients, and 30.7% of group 1 achieved ACR50 compared with 14.3% of group 2. DAS28 values of less than 2.6 were obtained in 15.4% of infliximab-treated patients compared with 7.1% of etanercept-treated patients. DAS28 percentage changes from baseline were -30.8 in group 1 and -16 in group 2. The percentages of patients with an HAQ score decrease of greater than 0.22 were 61.5% and 14.3% in groups 1 and 2, respectively [6] (3b level of Oxford evidence).

### Rate of efficacy (gaining major responses) in adalimumab-treated patients who failed treatment with infliximab or etanercept or both

In the ReAct (Research in Active Rheumatoid Arthritis) trial, Bombardieri and colleagues [7] evaluated the effectiveness and safety of adalimumab in RA patients who previously discontinued TNFα antagonists for any reason. Of 6,610 patients enrolled in the ReAct trial, 5,711 had never been treated with an anti-TNFα agent, 591 were previously treated with infliximab, 188 were treated with etanercept, and 120 were treated with both TNFα agents. In the infliximab group, 507 patients underwent adalimumab treatment, and 22% discontinued due to no response, 51% due to loss of response, and 27% due to intolerance. In the etanercept group, 151 patients received adalimumab, and 42% discontinued due to no response, 32% due to loss of response, and 26% due to intolerance. At week 12, 60% of patients switched to adalimumab had an ACR20 response and 33% had an ACR50 response; 76% had a moderate and 23% had a good EULAR response. In addition, 12% achieved a DAS28 of less than 2.6 and 13% achieved an HAQ score of less than 0.5 [7] (2b level of Oxford evidence).

Nikas and colleagues [8] confirmed the previous data showing that adalimumab has the same efficacy in 25 RA patients naïve to biological treatment and 24 who had previously used infliximab. After 1 year of follow-up, clinical improvement was similar in the two groups: ACR20, ACR50, and ACR70 responses were achieved by 75%, 50%, and 33% of switchers, respectively [8] (3b level of Oxford evidence).

### Rate of efficacy (gaining of major responses) in rituximab-treated patients who failed treatment with TNFα blockers

In the REFLEX (Randomized Evaluation of Long-Term Efficacy of Rituximab in RA) trial, Cohen and colleagues [9] evaluated primary efficacy and safety at 24 weeks in patients with active RA and an inadequate response to one or more anti-TNFα agents. Three hundred eleven patients received a course of rituximab while 209 patients received placebo. At week 24, ACR20, ACR50, and ACR70 responses were achieved in 51%, 27%, and 12%, respectively, in the group treated with rituximab compared with 18%, 5%, and 1% in the placebo group. Rituximab-treated patients showed a trend toward less progression in radiographic endpoints, and all ACR response parameters were significantly improved. Clinical improvement was seen in fatigue, disability, and health-related quality of life (demonstrated by FACIT-F [Functional Assessment of Chronic Illness Therapy-Fatigue], HAQ disease index, and SF-36 [Short Form Health Survey-36] scores, respectively) [9] (1b level of Oxford evidence). Moreover, as shown by Finckh and colleagues [10], the switch to rituximab after the failure of the first anti-TNFα seems to be more effective than switching to the second anti-TNFα agent.

### Rate of efficacy (gaining of major responses) in abatacept-treated patients who failed treatment with TNFα blockers

In the Abatacept Trial in Treatment of Anti-TNF Inadequate Responders (ATTAIN), abatacept was studied in RA patients who failed anti-TNFα therapy. Three hundred ninety-one patients were followed for 6 months. ACR20, ACR50, and ACR70 responses were 50.4%, 20.3%, and 10.2% in the treated group compared with 19.5%, 3.8%, and 1.5% for those receiving placebo. DAS28 remission rates were 10.0% in the treated group and 0.8% in the placebo group [11] (1b level of Oxford evidence).

In the ARRIVE (Abatacept Researched in RA patients with an Inadequate anti-TNF response to Validate Effectiveness) trial, which aimed to assess the safety and tolerability in patients with active RA who had failed up to three anti-TNFα agents, the mean reduction in DAS28 at 6 months was -2.0 from baseline. Overall, 22.4% of patients achieved a low disease activity state and 13.0% achieved remission [12] (1b level of Oxford evidence).

### Rate of efficacy (gaining of major responses) in tocilizumab-treated patients who failed treatment with anti-TNFα

In the RADIATE (Rheumatoid Arthritis Study in Anti-TNF Failures) trial, Emery and colleagues [13] recently showed the improvement in 499 RA patients who had inadequate response to at least one anti-TNFα antagonist and who were treated with tocilizumab. DAS28 remission (DAS28 of not more than 2.6) rates at 24 weeks were clearly dose-related, being achieved by 30.1%, 7.6%, and 1.6% of 8 mg/kg, 4 mg/kg, and control groups (*P *= 0.0001 for 8 mg/kg and *P *= 0.053 for 4 mg/kg versus control). Both 8 mg/kg (50.0%) and 4 mg/kg (30.4%) groups had a higher rate of ACR20 response versus control (10.1%; *P *< 0.0001). ACR50 and ACR70 responses after 24 weeks were achieved by 28.8% and 12.4% of patients in the 8 mg/kg group (*P *< 0.0001 and *P *< 0.0002, respectively, versus control) [13] (1b level of Oxford evidence).

## Discussion

Several studies analyzed the efficacy and the gaining of major responses in RA patients treated with different biological drugs. Table 1 summarizes the results obtained from our research. Few studies had strong evidence, mainly evaluating efficacy in switching from anti-TNFα to new targeted therapies (rituximab, abatacept, and tocilizumab). The data available suggest that many of the clinical considerations and decisions that we adopt rely on less than strong scientific evidence. Clinicians should consider the data we present as a practical approach in making the everyday decisions to reach the lowest possible level of clinical activity [14].

The data show that switching from infliximab to etanercept is effective, with an ACR20 response of between 33% and 64% according to different studies. Moreover, etanercept seems to be able to maintain the ACR20 response obtained with infliximab when the latter biological agent is stopped because of side effects [15]. Data show that a previous failure with etanercept does not influence the response to infliximab with a reach of clinical response. Indeed, there is no doubt that a clinical effect can be reached by switching.

Switching to another biological agent in patients treated with infliximab is due mainly to a loss of response during treatment, whereas in patients treated with etanercept, switching is due to non-response. The effectiveness of adalimumab seems not to be influenced by previous treatment with infliximab.

In regard to patients treated with a second TNFα inhibitor who failed the first, data show a continuation rate of a second TNFα blocker of 73% of switchers after 15 months of follow-up [16]. Switchers to a second anti-TNFα drug seem to have high treatment rates of continuation, and first drug withdrawal due to inefficacy is associated with an increased rate of a second drug withdrawal due to inefficacy but not toxicity; similarly, first drug withdrawal due to toxicity is associated with an increased rate of a second drug withdrawal due to toxicity but not inefficacy. All of these data suggest that a clinical response can be obtained from switching, but given the high cost of the drug, it seems questionable to rely on this response value as a way of treating all poor responders (not reaching low disease activity or DAS remission). In fact, in terms of major outcomes such as DAS remission and ACR70, the results are less convincing. Not more than 5% to 15.4% of the patients overall can be led to remission. This was confirmed in the last trial with golimumab recently published, that showed the 10% rescue to the major response [17]. This is clearly disappointing when facing the first failure.

In clinical practice, it is reasonable to switch from one anti-TNFα agent to a second one, possibly with a different mechanism of action. It does not appear useful to switch to a third one [18], but it seems worthwhile to treat patients who are resistant to this therapeutic approach with other biological agents, such as anti-CD20, abatacept, or tocilizumab.

Rituximab has been said to be more effective than switching to an alternative anti-TNFα agent. Moreover, it was demonstrated that treatment with rituximab may be more effective than switching to an alternative anti-TNFα agent in RA patients in whom active disease persisted despite anti-TNFα therapy [10], even though the difference seems to be really more statistically than clinically significant. Similar data have been seen in clinical trials that have confirmed the efficacy of switching to abatacept from an anti-TNFα agent, thus suggesting that using a drug with a different mechanism of action may help.

In RA patients who do not respond to the anti-TNFα strategy, another therapeutic option is tocilizumab with a dose-dependent clinical response with a strong discrepancy between DAS remission and ACR70. Data suggest that a strong effect on pain and acute-phase reactants leads to this dissociation. However, comparing the changes in disease activity and response to tocilizumab using the Clinical Disease Activity Index (CDAI) and Simplified Disease Activity Index (SDAI) in three clinical trials (LITHE, OPTION, and TOWARD), Smolen and colleagues [19] recently found significant differences in changes of CDAI and SDAI between treatment with tocilizumab and placebo, with a minimal impact of C-reactive protein on clinical disease activity measures.

The introduction of anti-TNFα marked the beginning of a new era in the treatment of RA. Nonetheless, the efficacy of each biological agent is not similar in all patients, underlying the need for a drug switch between the agents. Moreover, the switch to different biological target therapies could be necessary because of the presence of driving cytokines other than TNFα. In general, an ACR20 can be obtained in 60% or more of the poor responders to the TNFα blockers, and an ACR70-DAS remission value can be obtained in roughly 10% to 15% of the cohorts after the TNFα blockade, with either CD20 depletion or CTLA4 stimulation. The rule of a 10% to 15% gain of effect after TNFα, the major target, seems to be a good, but not ideal, perspective for the future of RA. These data strongly argue for a clear-cut need for biomarkers capable of identifying, at baseline, which RA patients having different pathways will be the best responders to some biologics or poor responders to other biologics. Yet these biomarkers are not at hand. Furthermore, prescribing a biological agent is an important decision that can greatly impact the quality of life of a patient and can be associated with varying medical costs, owing to differences in routes of administration (depending on the agent). Etanercept and adalimumab are administered subcutaneously. Both can be self-administered at home, but they have some disadvantages, including the requirement of more frequent administration and the risk of pain and local site reactions. Intravenous drugs can be inconvenient for patients with respect to traveling and taking time off from work for infusions and also for patients with difficulties regarding intravenous access. Intravenous administration may use more health care resources and require infusion rooms and equipment as well as medical and nursing supervision [20]. Anti-TNFα agents require a short time of administration to reach their effect, and anti-CD20 and abatacept have to compete with this very practical issue that needs to be considered when a choice has to be made to reach a major response. In addition, the more frequent administration with some TNFα blockers, required to better control the disease activity status, should lead to a careful analysis of cost in all of these situations.

## Conclusions

The efficacy of different biological agents in selected randomized controlled trial populations is not similar in all patients, underlying the presence of different pathways driving the inflammatory process. The rescue to a major response seems to occur at a rate of 10% to 15% among the various biologics. Annual costs and administration modalities need to be taken into account when making therapeutic decisions in non-responders.

## Abbreviations

ACR: American College of Rheumatology; ACR20: American College of Rheumatology 20% improvement criteria; ACR50: American College of Rheumatology 50% improvement criteria; ACR70: American College of Rheumatology 70% improvement criteria; CDAI: Clinical Disease Activity Index; DAS: disease activity score; DAS28: disease activity score using 28 joint counts; EULAR: European League Against Rheumatism; HAQ: Health Assessment Questionnaire; RA: rheumatoid arthritis; SDAI: Simplified Disease Activity Index; TNFα: tumor necrosis factor-alpha.

## Competing interests

GF has received consulting and speaking fees from Abbott Laboratories (Abbott Park, IL, USA), Wyeth (Madison, NJ, USA), Roche (Basel, Switzerland), and Bristol-Myers Squibb Company (Princeton, NJ, USA) (less than €10,000 each). The other authors declare that they have no competing interests.

## Authors' contributions

SA, AL, and GF conceived of the study and participated in its design and coordination, in data acquisition and analysis, and in manuscript preparation. EG and AZ participated in data acquisition and in manuscript preparation. All authors read and approved the final manuscript.
